# Differential response by tandem leaders and followers to landmark-rich and landmark-poor environments

**DOI:** 10.1007/s10071-025-01958-x

**Published:** 2025-05-19

**Authors:** Norasmah Basari, Ana B. Sendova-Franks, Alan Worley, Nigel R. Franks

**Affiliations:** 1 School of Biological Sciences, Bristol Life Sciences Building, University of Bristol, 24 Tyndall Avenue, Bristol, BS8 1TQ UK; 2 Present Address: Faculty of Science and Marine Environment, Universiti Malaysia Terengganu, Kuala Terengganu, Malaysia

**Keywords:** Ant, *Temnothorax albipennis*, Tandem running, Route familiarity, Visual information, Navigation

## Abstract

When animals use the same route repeatedly, they have the opportunity to update information that might help them to navigate more quickly and more accurately. Here we analyse ants involved in tandem running, in which the leader has evaluated a new nest and decided to recruit to it while the follower has chosen to be led and shown the route. We used a motorised gantry equipped with a camera to track the movements of tandem members on their tandem and return trips in a landmark-rich and a landmark-poor environment. Although the amount of visual navigational information did not affect the movements of leaders or followers on their tandem trip, the paths of followers were significantly more tortuous and their speeds significantly slower than those of leaders on their return trips in the landmark-poor environment. By contrast, there were no such differences between the followers and leaders on their return trips in the landmark-rich environment even though the return paths of followers in the landmark-rich environment were significantly more tortuous than that of leaders in the landmark-poor environment. Indeed, in the landmark-rich environment, the majority of the leaders’ return paths had loops while most were straight in the landmark-poor environment. Thus, the availability of more information when many landmarks are present may induce tandem leaders to make the loops, typically associated with the paths of tandem followers. This suggests knowledgeable individuals slow down to update navigational information and has implications for the formation of leader oligarchies in tandem running.

**Supplementary Information:**

The online version contains supplementary material available at 10.1007/s10071-025-01958-x.

## Introduction

Animal navigation has been a research topic of general interest to students of animal behaviour and beyond for many years (Waterman 1989; Lehrer 1997; Gould 1998, 2015; Frost and Mouritsen 2006; Gould and Gould 2012; Mueller et al. 2013; Wiltschko and Wiltschko 2022; Jeffery et al. 2024). A fundamental question is how animals extract and use information from their surroundings to find their way home. Animal navigation is well studied for vertebrates such as birds, mice, squirrels, monkeys, turtles and other marine dwellers (Domjan 1987; Sutton et al. 2000; Åkesson et al. 2003; Alerstam et al. 2003; Vlasak 2006; Wilson et al. 2015; see also review in Bingman and Cheng 2005). Vertebrates have much bigger brains than invertebrates and hence it may be tempting to assume vertebrates are able to solve greater navigational challenges. However, detailed studies on navigation in invertebrates such as ants and bees provide evidence that small-brained animals are also able to extract information from the environment and solve complex tasks in their navigation (Collett 1996; Merkle and Wehner 2008; Legge et al. 2010; Wystrach et al. 2011; Schultheiss et al. 2013; see also Wystrach and Graham 2012 for a review). Indeed, recently it was demonstrated that social learning influences communication with reference to spatial orientation in bees as it is known to do in vertebrates (Dong et al. 2023).

Here, our aim is to investigate whether animals that move frequently to and from their nests take further advantage of navigational information even though they are knowledgeable about the target and the route to it. Previous studies on both vertebrates and invertebrates (Lehrer 1993; Graham and Collett 2006; Guilford and Biro 2014) have shown that the speed of landmark recognition and the recall and utilization of information increase when animals use the same route repeatedly. For example, it has been demonstrated experimentally that pigeons reduce deviations from the straight line of their homing routes as they become familiar with landmarks in the environment (Guilford and Biro 2014). This suggests that animals are continually updating the navigational paths they use every time they move to and from their home, as they become familiar with the route.

In ant colonies with small populations such as those of *Temnothorax albipennis*, a one-to-one recruitment strategy, known as tandem running, is used to teach other naïve members about the location of a resource, such as a new nest or a food source (Franks and Richardson 2006). This one-to-one recruitment requires the tandem-leading ant to have enough information to teach the naïve ant (the follower) the route to the resource. During tandem running, the leader ant pauses a few times to allow the follower ant to learn the route (Franks and Richardson 2006; Franklin et al. 2011; Basari et al. 2014a). This imposes a cost on the leader because it can proceed four times faster when not encumbered by a follower (Franks and Richardson 2006). Many of the follower ants will become leaders in a subsequent tandem run (Richardson et al. 2018). This strongly suggests that the followers learn the route and acquire navigational information during tandem running (Franks and Richardson 2006).

In tandem running *T. albipennis* ants, the leader is knowledgeable about the route to the target because she has evaluated the resource and made the decision to recruit to it after having learned the route individually (as in the present study) or followed another leader. By contrast, the follower has chosen to be led and is naïve about the resource and the route to it (Franks and Richardson 2006; Franklin and Franks 2012). Indeed, after experimental interruption of tandem runs to a new nest, the majority of *T. albipennis* leaders proceed to the target while the majority of followers return to the old nest after an extensive search, even though there is some evidence to suggest such incomplete tandem runs provide some directional information to the former followers (Franks et al. 2010; Basari et al. 2014a). Interrupted followers in the ponerine ant *Diacamma indicum* need guidance from nestmates to be able to reach the new nest (Kaur et al. 2017) but recent studies have demonstrated functional and mechanistic differences between tandem running in *Temnothorax* and *Diacamma* (Mizumoto et al. 2023). On the other hand, in the Australian ant *Camponotus consobrinus*, there is evidence that the followers in tandem runs to a food source are often experienced foragers (Schultheiss et al. 2015). This is probably because the food source is on a complex structure, namely a tree in the field, and individuals that had visited one food source were guided to a new different food source on the same tree (Schultheiss et al. 2015).

Tandem running in *Temnothorax* ants represents a combination of social and private information about the route to a target and its value as a resource (Stroeymeyt et al. 2017). Recent evidence shows that navigational information to a food source can spread through sequential social learning in 90% of tandem runs in *Temnothorax nylanderi* colonies where followers that become leaders in turn guide their followers along the route they themselves have been shown (Colomer-Vilaplana et al. 2025). On the other hand, independent exploration, after the tandem run, improves the performance of a former tandem-run follower as a tandem leader to a new nest; her subsequent tandem path is less tortuous than that of a former follower that has not performed individual exploration (Franklin and Franks 2012). The leader may also keep on updating her own route information while leading a tandem run or indeed on her return journey. For instance, Richardson et al. (2018) have shown that during repeated nest emigrations, certain ants are particularly successful leaders of tandem runs, and through a positive-feedback process, they form a stable oligarchy, which controls the flow of information during house-hunting. One possible underlying mechanism is that even individuals who are already knowledgeable about the route to the target use navigational information to improve further. Indeed, route learning by individual ants can improve with experience (Harrison et al. 1989; Franklin and Franks 2012).

Here we ask whether *T. albipennis* tandem leaders and followers respond to the abundance of visual information available along the tandem route and whether the responses by such knowledgeable and naïve ants differ. We test them with regard to the route between the current, old, nest and the tandem-run target, namely a new nest. We use two treatments: (1) a Landmark-rich (L-R), and (2) a Landmark-poor (L-P) environment. We employ a motorised gantry equipped with a camera to track the ants on their tandem trips to the new nest and their return trips back to the old nest. Our main aim is to find out the effect of the visual navigational information in the L-P and L-R environment on the return trip because this is when the former leader and follower have to find their own way home to the old nest.

In particular, we expect the extent of visual navigational information to have some effect on the return trip of the follower, who has been led to the new nest — but how about the leader? Is the leader affected too, despite possessing enough knowledge to have led a tandem to the new nest? More specifically, if leaders are learning navigational information on the return trip, we expect their return paths to have similar loops to those of followers. Therefore, we expect leaders in the L-R environment to have higher path tortuosity and lower mean speed than leaders in the L-P environment. We expect followers to be learning on their return trip in both environments even if it might be easier for them to find their way back to the old nest in the environment richer in navigational information. Indeed, the desert ants *Cataglyphis fortis* from the harsh salt flats in Tunisia build their own landmarks in order to increase their chances of successful homing when the environment is completely featureless (Freire et al. 2023). Lastly, since leaders are more knowledgeable about the route in both environments, we expect leaders to have lower path tortuosity and higher mean speed than followers irrespective of the environment. The interdependence of the above relationships means that the difference between leaders in the L-P environment and followers in either the L-P or the L-R environment should be the largest both in terms of tortuosity and mean speed. Therefore, these differences are most likely to be significant.

The tandem trip is of secondary interest in relation to our main question. The follower in a *T. albipennis* tandem run loops during pauses to learn landmarks and the leader has to wait for the follower to catch up. The movements of both are dominated by the loops made by the follower. Nevertheless, we want to investigate whether the extent of visual navigational information in the L-P and L-R environment makes a difference to the tandem trips.

We keep the comparisons between return trips and the comparisons between tandem trips separate. Tandem trips are both tortuous and slow because there is a lag, and occasionally, a break, in the communication between the leader and the follower, resulting in a rather convoluted path (Franks and Sendova-Franks 2013). By contrast, when moving on their own, as on the return trip, individual ants move much more quickly on less convoluted paths (Franks and Richardson 2006). Hence, differences are to be expected between tandem and return trips. Furthermore, even though *T. albipennis* tandem followers learn landmarks that they can use on their return trip (Franks and Richardson 2006), the learning mechanisms employed during the tandem trip might be different from those used on the return trip. For example, it is possible that foraging ants from other species use different learning mechanisms for journeys from and to the nest (Collett and Collett 2000; Collett and Graham 2004) even though they can use visual cues during their outward journeys to guide them during their homeward (return) journeys (Graham and Collett 2006; Sakiyama and Gunji 2013).

Our experimental methodology ensures that each tested ant encounters the treatment environment for the first time, has no prior experience as either a tandem leader or a tandem follower in that environment and has an equal opportunity for acquiring environmental information prior to a tandem run.

## Materials and methods

We collected 12 colonies of the ant *T. albipennis* from the Dorset coast, England, UK on 14th March 2013. The experiments commenced on 15th April 2013 and were completed on 15th May 2013. In the laboratory the colonies were housed in standard nests consisting of a cardboard perimeter, sandwiched between two microscope slides (75 × 50 mm). The cardboard formed an inner nest cavity (measuring 50 × 35 × 2 mm) with a 2 mm-wide entrance. The colonies had *ad libitum* access to water, honey solution and *Drosophila* flies, which were replenished weekly. The ant colonies were kept in square (100 × 100 mm) Petri dishes and cardboard was placed on top of colony nests to make them dark between experimental trials (one trial was defined as the emigration of a single colony from its current nest to a new nest, see later).

**Fig. 1:**
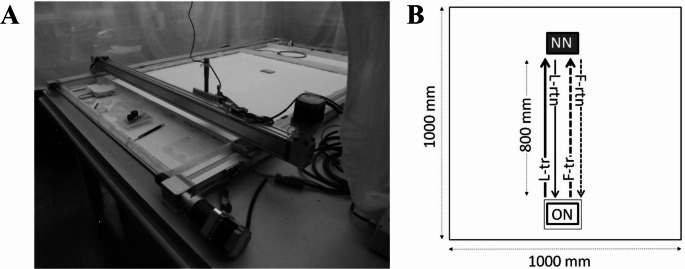
The experimental set-up. **A** Experiments were conducted in a 1000 × 1000 mm arena with a wall 40 mm high (centre). A motorised gantry (metal frame and crossbar) equipped with a camera was employed to track the ants on their tandem-run and return trips. The white muslin cloth, suspended above a strip light, so not to cover it from below, surrounded the arena during the Landmark-poor (L-P) treatment but was lifted during the Landmark-rich (L-R) treatment (see Methods for more detail). The rectangular piece of cardboard at the far end of the arena indicates the position of the new nest (NN). **B** The experimental arena was set up in the same way for the L-R and the L-P treatments. The NN was placed 800 mm away from the front of the old nest (ON). The rectangular box around the ON depicts the Petri dish used to cover the ON during the tracking of each tandem run. L-tr: leader’s tandem run path; L-rtn: leader’s return path; F-tr: follower’s tandem run path; F-rtn: follower’s return path. The return paths of former leaders and followers were tracked with the motorised gantry in alternate tandem runs

The experiments were carried out in an arena of 1000 × 1000 × 40 mm and involved two treatments: a Landmark-rich (L-R) and Landmark-poor (L-P) environment. Under L-R, the ants had access to prominent landmarks present around the arena, such as computers behind the old nest and lab instruments on the right side of the arena (relative to the old nest), and to celestial cues on the left side of the arena through the uncovered lab windows. The lab lights were switched on and there was a strip light above the experimental arena. Under L-P, the ants were barred from access to prominent landmarks and celestial cues because the arena was surrounded with white muslin cloth (Fig. 1A) and the windows were covered with blinds. The lab lights were switched on and the strip light above the experimental arena was visible from below because the muslin was suspended over it. The muslin fell behind the arena walls on all four sides without obscuring them (Fig. 1A). Ants were not familiar with the landmarks around the arena because colonies were housed in a different area of the lab and were only brought to the experimental arena prior to the start of an experimental trial involving a colony emigration to a new nest.

### Experimental protocol

At the start of each trial for each of the L-P and L-R treatments, the old standard nest (ON) was downgraded by having its top removed to induce emigration. A new standard nest (NN) was placed 800 mm away from the front of the ON. When a tandem run began, the other remaining ants in the arena were removed and placed in a separate Petri dish until the end of the trial. The ON was temporarily isolated by placing a Petri dish lid over it to prevent other ants making contact with the tandem pair and stop the formation of other tandems. The tandem pair was tracked with a motorised gantry (Fig. 1A), including a mounted USB camera (Basari et al. 2014a), until they reached the new nest (Fig. 1B, L-tr). Using the gantry, we recorded the tandem run and either the returning leader or the returning follower alternately for each tandem run. During a tandem run, an operator used a joystick to move the gantry and track the leader and follower ants simultaneously by observing their progress on the image relayed by the gantry camera to a software interface window on a PC. The coordinates of the ants’ centroids were recorded automatically in separate pairs of columns and stored in an output file at the end of the tracking for that trip. If either of the ants went outside the camera’s field of view, the tracking of that tandem run was aborted. To determine which coordinates belonged to the leader and which belonged to the follower, the program used the values of the coordinate representing the direction of tandem-run travel – the positive direction of the y-axis here. The ant with the higher values for the y-coordinate was the leader. The leader/follower allocation could be imperfect under rare circumstances. We estimate the error rate as 2% because this was the percentage of pairs of points where the y-coordinate of the leader was lower rather than higher than the y-coordinate of the follower. We matched the return path for the leader or follower with the respective tandem-run path. Therefore, the quoted Ant ID is associated with the tandem and return paths of either a leader or a follower.

If the leader was tracked on the return trip, after the pair had reached the NN, we removed the follower when there was a gap between the two ants. This was done carefully so as not to disturb the leader. By removing either the leader or the follower ant we have no need to mark the trial ant. At the scale of a 1000 × 1000 mm arena, it is virtually impossible to follow individually marked ants that are 2–3 mm long and track them again in subsequent tandem and return trips. On this scale it is also difficult to keep track of more than one individual once they have reached and entered the NN. The leader ant was left to access the NN alone and was tracked on its return trip to the ON (Fig. 1B, L-rtn). Recording started from the NN entrance and continued until the ant was considered to have successfully returned to the ON (see later). The tracked ant was then removed from the arena and kept in a separate Petri dish (as was the follower) until the end of the trial. The Petri dish cover over the ON was lifted again to allow another tandem run to form.

If the follower was tracked on the return trip, the ants were tracked during tandem running from the ON until they reached the NN (Fig. 1B, F-tr). When the pair had reached the NN, the leader ant was removed and only the former follower ant was allowed to access the NN. After that, the former follower ant was tracked as it returned from the NN back to the ON (Fig. 1B, F-rtn). It was then removed from the arena and kept in a separate Petri dish (as was the leader) until the end of the trial.

A tandem was considered to have reached the NN when the pair was very close to it but it was still safe to remove the ant that was not being tracked without disturbance. Occasionally, the tandem pairing broke down near the NN, but as long as the tracked leader or follower entered the NN, we considered the tandem run to have been successful. We considered an ant to have successfully returned to the ON if it had reached y = 0 mm, namely the y-coordinate of the ON’s entrance, at any value of x. We could not always continue to follow the ant to the entrance of the ON with the gantry because some ants walked near the arena perimeter before they entered the ON and this could cause image-analysis issues with the arena wall.

The removal of the tracked ants from the arena and their isolation from the colony for the duration of an experimental trial avoided pseudoreplication. In other words, we ensured that the same ant (leader or follower) was tracked only once during the same trial: on its paired tandem and return trips. Furthermore, the removal of all other ants in the arena and their isolation from the colony for the duration of the experimental trial ensured that the tracked ants had an equal opportunity to explore the arena prior to the tandem run and had no prior experience either as a leader or as a follower during that trial. We did not measure the duration of arena exploration by each ant prior to the tandem run but the differences in the duration of such prior experience would have been minimised by our procedure of tracking the first tandem run that formed after the ON was downgraded or the Petri dish covering the downgraded ON was lifted.

After the end of each colony emigration (trial), all the isolated ants, those that had taken part in the tracked tandem runs as well as all other ants in the arena, were reunited with their colony in the old nest after replacing its top slide and putting it into a Petri dish, and the arena floor was cleaned using alcohol and water to remove any traces of pheromone trails.

The L-R treatment was performed first on all 12 colonies and a day after it was completed the L-P treatment was performed on all 12 colonies. This was done to keep the environmental set-up as similar as possible for all trials under the L-R treatment. There was at least a 6-day gap before the same colony was used again for the L-P treatment. This period has been shown to be sufficiently long for *T. albipennis* ants to forget their emigration experience (Langridge et al. 2004). Even though we carried out a trial with each of the colonies under each of the two treatments, not all 12 colonies contributed to the data for each treatment: in some trials colonies either did not emigrate to the new nest or the tandem runs broke off before they reached the new nest. Overall, we analysed data from 10 colonies under the L-R treatment and eight colonies under the L-P treatment. In total, 64 pairs of tandem-run and return paths were recorded: 15 for leaders and 17 for followers under the L-R treatment and 15 for leaders and 17 for followers under the L-P treatment (Table S1).

It was not possible to record the data blind owing to the nature of the treatments. However, all the data were quantitative and the tracking system ensured that the coordinates were correctly recorded irrespective of the treatment conditions.

### Statistical analysis

Coarse graining was performed on the raw data to smooth any spurious turns along the ant paths. As the motorized gantry recorded the ant coordinates every 0.05s, there were quantization effects whereby ants could only move into one of the eight neighbouring pixels within the short sampling interval (Basari et al. 2014a). This resulted in some spurious turning angles. We analysed the tortuosity and speed of the tandem-run and return paths for leaders and followers after such coarse-graining (Basari et al. 2014a).

We performed the coarse graining using a bespoke program written in the R statistical package v. 4.3.2 (R Core Team 2023). The raw paths of the ants were smoothed by conducting three iterations of two-times coarse graining, each reducing the number of path points by half. This is equivalent to an overall eight times of coarse graining (for more details see Basari et al. 2014a). Since the original paths were recorded every 0.05s, such coarse graining resulted in a time interval of 0.4s for the analysed paths. Path tortuosity was calculated as $\:1-\frac{straight\:line\:distance}{total\:distance}$ and higher values indicate greater tortuosity. Speed was calculated as the average instantaneous speed over the whole path. We also identified, by eye, paths as containing loops if the path went over itself at least once without touching the NN or the Petri dish covering the ON.

All statistical analyses and graphical representations were carried out in the R statistical package v. 4.3.2 (R Core Team 2023). We used bespoke scripts including functions from the packages ggplot2 (Wickham 2016) and lattice (Sarkar 2008) for graphics, lme4 (Bates et al. 2015) for Linear Mixed-effects Models (LMMs) and multcomp (Hothorn et al. 2008) for post-hoc tests between means, and spatstat.geom (Baddeley et al. 2015) for calculating distances between pairs of tandem and return paths (see below).

#### Path tortuosity, path median x-coordinate and mean speed

To test for any differences in movement characteristics between tandem Leaders and Followers, we fitted a General Linear Mixed Model (GLMM) to path tortuosity and another GLMM to mean speed according to, in each case, Treatment (Landmark-rich or Landmark-poor), Role (Leader or Follower) and Trip (Tandem or Return, *N* = 128).

After plotting all Tandem and Return paths, we observed that they were biased to the right side on the Return trip of Leaders and Followers in the L-P Treatment. We used the median x-coordinate of the path as a measure of this bias. Given that the entrances of the ON and the NN were at x = 500 mm, an unbiased path between them is expected to have a mean x-coordinate close to 500 mm and an equal spread on both sides of that value. We tested for any effect of the three factors Treatment, Role and Trip on the path position around x = 500 mm by fitting a full GLMM.

The full GLMM model for each of tortuosity and mean speed included Treatment, Role, Trip, the three binary and one tertiary interaction between the three fixed-factor predictors. Colony with Ant ID nested within Colony were included as random-factor predictors to account for the repeated use of colonies and the paired nature of the tandem and return trips associated with the same ant. We used the AIC-minimisation criterion to select the best model for each of the two response variables. In each case, we compared the full model with the random-factor predictor Ant ID nested within the random factor Colony, the full model with each random factor separately, a reduced model with the tertiary interaction of the fixed factors excluded and only the random factor Ant ID included, and a full General Linear Model (GLM), which did not include any random factors.

For each of the response variables tortuosity and mean speed, the GLMM with the random factor Ant ID nested within the random factor Colony and the GLMM with the random factor Colony on its own showed signs of overfitting (singularity). The remaining three models for each of tortuosity and mean speed had a similar AIC with a difference of less than 2. In order to capture the full treatment structure and the repeated nature of the experimental design, the final model for each of tortuosity and mean speed included the three fixed-factor predictors with all possible interactions between them and the random-factor predictor Ant ID randomly varying around the global mean.

For the response variable median x-coordinate of the path, we applied a model with the same structure as the final models for tortuosity and mean speed. Our justification was the same. We wanted to capture the full treatment structure and the repeated nature of the experimental design, and the difference in AIC with the other four considered models was less than 2.

For the response variable tortuosity, the difference between the final model and the GLM was not significant (χ^2^ difference = 1.11, d.f. = 1, *p* = 0.293). This means that taking the random factor Ant ID (repeated measures for the same ant on the tandem and return trip) into account did not make any difference to the model quality. We still used the mixed model because of the paired nature of the experiment and because the difference in AIC between the two models was less than 2. The final model fitted the data adequately (range of scaled residuals: -1.72 to 1.97, Shapiro-Wilks Normality test for scaled residuals: W = 0.97, *n* = 128, *p* = 0.016, Fig. S1, Table S2A-E). Therefore, the residuals were within the expected range but the null hypothesis that they were normally distributed could be rejected at the 5% significance level due to some deviations from expectation at the very end of both tails (Fig. S1). This is not unusual with a sample size above 100.

For the a-posteriori measured variable median x-coordinate of the path, the difference between the final model and the GLM was significant (χ^2^ difference = 6.90, d.f. = 1, *p* = 0.009), which means that the inclusion of the random factor Ant ID was statistically significant. The final model fitted the data well (range of scaled residuals: -2.76 to 2.22, Shapiro-Wilks Normality test for scaled residuals: W = 0.99, *n* = 128, *p* = 0.537, Fig. S2, Table S3A-C).

For the response variable mean speed, the difference between the final model and the GLM was not significant (χ^2^ difference = 0.202, d.f. = 1, *p* = 0.653). The final model did not fit the data well (range of scaled residuals: -4.42 to 2.75, Shapiro-Wilks Normality test for scaled residuals: W = 0.92, *n* = 128, *p* = 9.211*10^− 7^, Fig. S3, Table S4A-C). Much of the poor model fit could be accounted for by an influential outlier, Ant 15, the only individual to have a lower mean speed on the return trip than on the tandem trip (Fig. 6, Treatment L-R, Role L). However, deviations from expectation remained at both tails of the distribution of model residuals even after the removal of the data for Ant 15 (Figs. S3). To err on the side of caution, we kept the data for Ant 15 in the model.

#### Distance between tandem and return paths

To calculate the distance between the Tandem (T) and Return (R) paths for an individual ant we used the nncross function from the spatstat.geom R package, which calculates the nearest neighbour (nn) distance for each point of one point pattern in the second point pattern. The T and R paths are likely to have different numbers of data points. For each data point on the T path, we found the nn distance using the points in the R path. Then we did the same for every point in the R path using the points in the T path. This is needed because there is not necessarily a reciprocity in nn distances; a T path point may have a nn R path point, yet that R path point’s nn is a different T path point. We did include the same nn distance twice when there was a reciprocity because this is the fairest representation: a nn distance for each possible nn distance pairing between the paths. Then we summed all the nn distances and divided this sum by the total number of data points (in the T and R paths together). If the two paths are identical, the result is 0 mm. Otherwise, it is a separation with, in principle, no upper limit. For comparison, the distance between the antennae tips is 1.77 mm (for body length of 2.88 mm, based on one image of a *T. albipennis* worker from the experimental tracking images).

To test for any evidence that tandem Leaders and Followers in the two treatments were following the Tandem path on their Return trip and whether they differed in that respect, we fitted a General Linear Model (GLM) to the log10-transformed distance between T and R paths according to Treatment (Landmark-rich or Landmark-poor) and Role (Leader or Follower, *N* = 64). We compared the full model with each of the three other possible models (both predictor factors without interaction and each factor on its own) and used the AIC-minimisation criterion to select the best model.

Although, the models containing either of the two predictor factors, Role or Treatment, on its own had the smallest AIC, we chose the full model as the final one, because it was more representative of the experimental design and its AIC differed by less than 2 from the AIC values of the above single-predictor models. Indeed, the difference between the final model and the GLM with Role as the single factor predictor (with the smallest AIC) was not significant (χ^2^ difference = 3.149, d.f. = 2, *p* = 0.207). The final model fitted the data well (range of scaled residuals: -2.05 to 3.01, Shapiro-Wilks Normality test for scaled residuals: W = 0.99, *n* = 64, *p* = 0.641, Fig. S4, Table S5A-D).

## Results

### Path tortuosity and path median x-coordinate

**Fig. 2:**
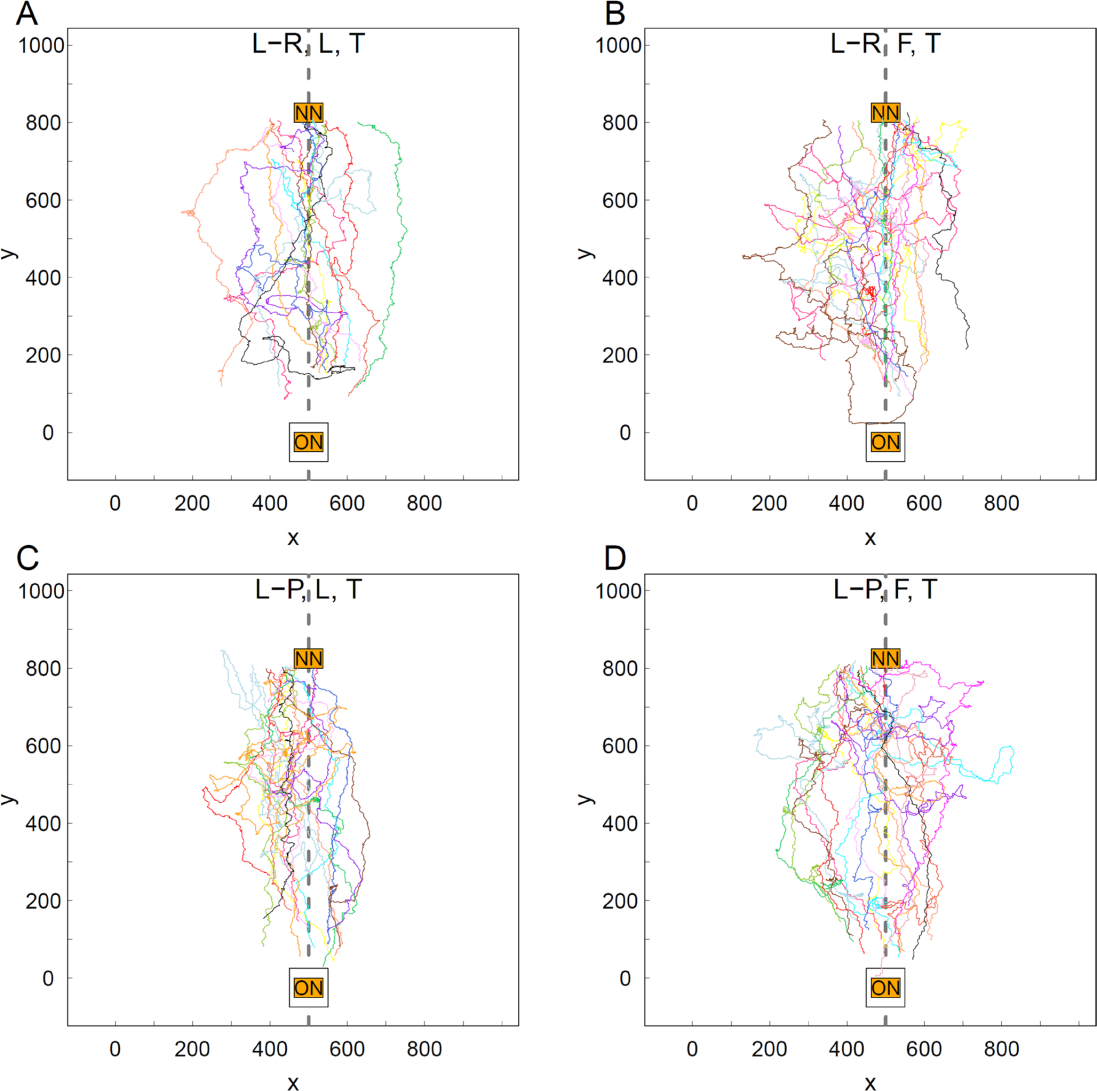
The paths of tandem members on the tandem trip. **A** Treatment Landmark-rich (L-R), Role Leader (L), *n* = 15. **B** Treatment L-R, Role Follower (F), *n* = 17. **C** Treatment Landmark-poor (L-P), Role L, *n* = 15. **D** Treatment L-P, Role F, *n* = 17. ON indicates the old nest with exit at x = 500 mm, y = 0 mm. NN indicates the new nest with exit at x = 500 mm, y = 800 mm. The black rectangle around the ON indicates the Petri dish covering the ON during the tracking of a tandem run by the gantry. The interrupted grey line indicates the midpoint along the x-axis, x = 500 mm. The colour of the tandem path here is the same as the colour of the return path for the same ant in Fig. 3

**Fig. 3:**
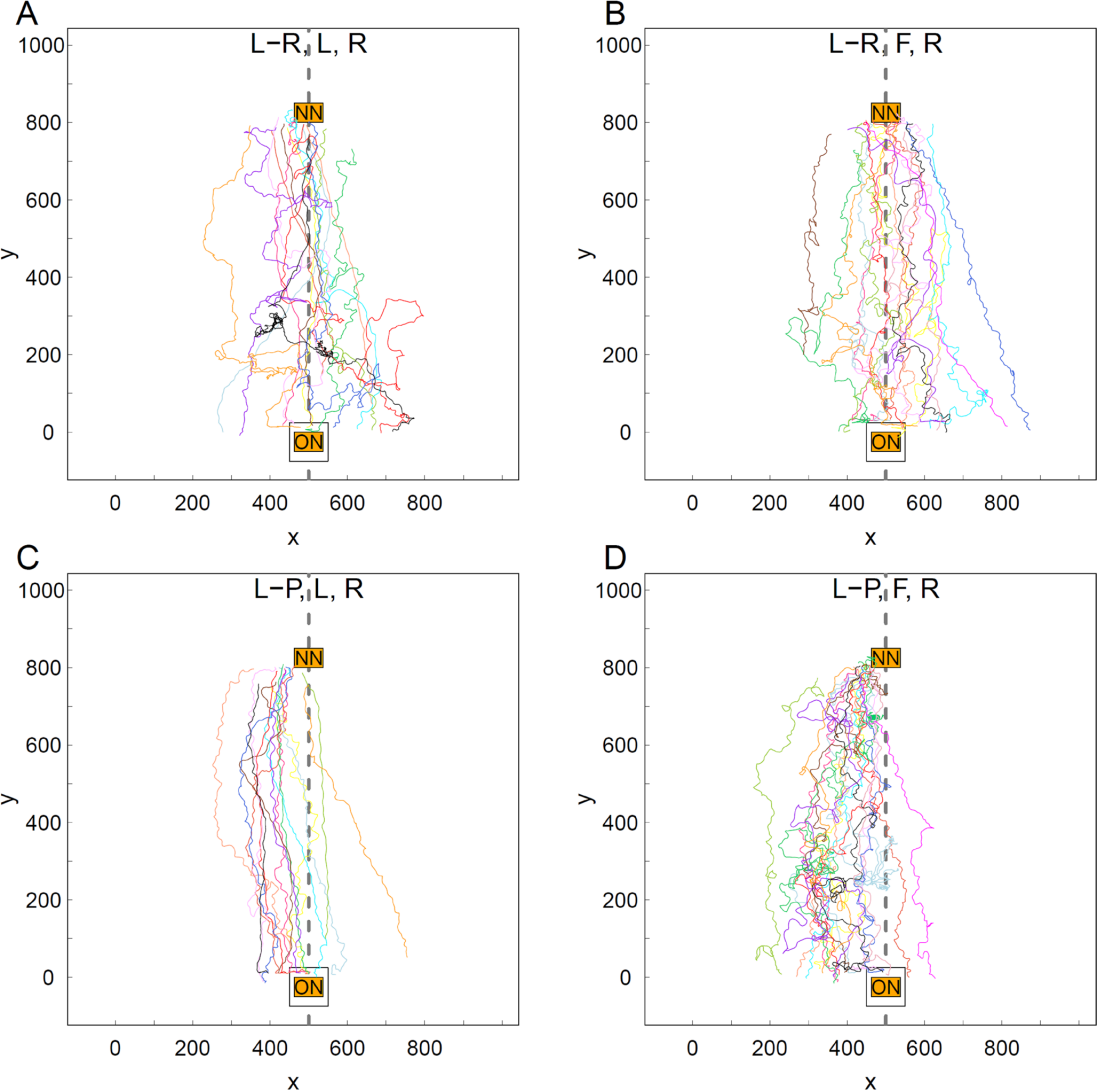
The paths of former tandem members on the return trip. **A** Treatment L-R, Role L, *n* = 15. **B** Treatment L-R, Role F, *n* = 17. **C** Treatment L-P, Role L, *n* = 15. **D** Treatment L-P, Role F, *n* = 17. For the meaning of abbreviations and symbols, and other details see the caption for Fig. 2

**Fig. 4:**
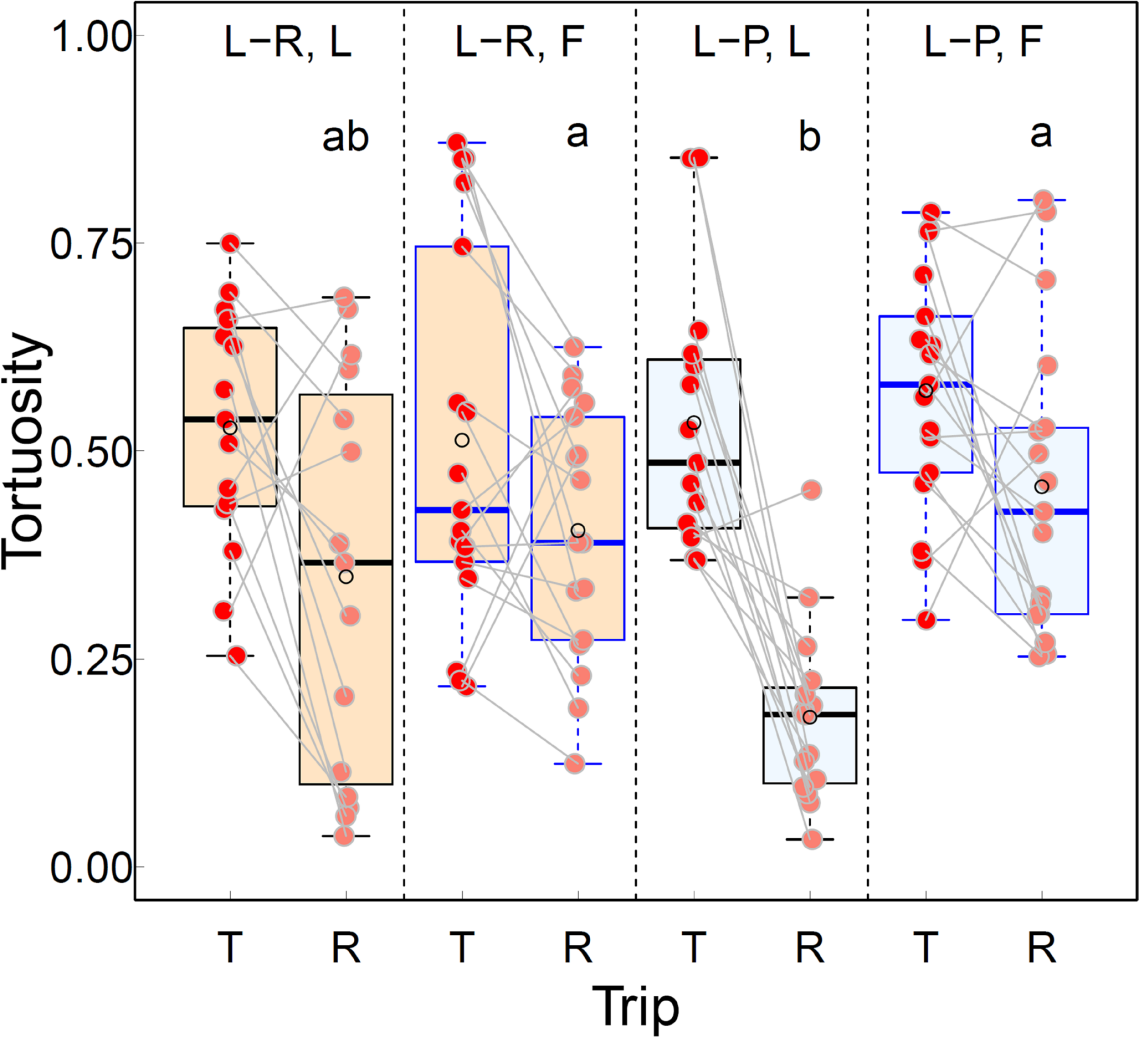
The effect of Treatment (Landmark-rich or Landmark-poor), Role (Leader or Follower) and Trip (Tandem or Return) on mean path tortuosity. The box encompasses the distance between the 25% and 75% quartiles, the interquartile range (IQR). The horizontal line in the box represents the median. The whiskers extend to the outermost values within 1.5 times the respective IQR. The superimposed empty black circle represents the mean and the superimposed filled circle is the individual measurement. The grey line connects the Tandem and Return values for the same individual ant. A small amount of jitter was applied to the filled circles to minimise any occlusion. L-R or L-P, L, T or R: *N* = 15, L-R or L-P, F, T or R: *N* = 17. Means for Return trips not sharing a letter are significantly different at the 5% significance level (GLMM followed by a post-hoc pair-wise comparison between means, Table 1). It was decided in advance to compare means between Tandem trips separately (see Introduction); no significant differences were found (Table S6)

The paths of individuals tended to be straighter on the Return trip than on the Tandem trip for both Roles (L and F) in both Treatments (L-R and L-P, Figs. 2 vs. Fig. 3, paired values for individual ants in Fig. 4). There were no significant differences in the tortuosity of Tandem paths in any of the pair-wise comparisons between Treatment × Role combinations (Fig. 4, Table S6). Indeed, virtually all paths on the Tandem trip contained loops along a wide range of sizes: from very large lasso-like ones to tiny knots (Figs. 2 and S5). This strongly suggests that, as expected, the tortuosity of the Tandem path was dominated by the movements of the Follower and that Followers in both environments had a lot to learn.

The Return paths of Leaders were remarkably straighter in the L-P environment than in the L-R environment (Fig. 3A vs. Fig. 3C). Indeed, in agreement with our expectation based on the assumption that Leaders could still be learning environmental information in the richer, L-R environment, the Return paths of nine out of the 15 Leaders in the L-R environment (Ants 2–3, 7–8, 10–11, 13–15, Fig. S5) contained loops while loops were present in the Return path of only one out of the 15 Leaders in the L-P treatment (Ant 37, Fig. S5). Thus, the odds ratio of loops being present in the Return path of a Leader in the L-R environment versus in the L-P environment was significantly different from 1 (Fisher’s exact test, two-tailed, estimated odds ratio = 18.72, 95% CI 1.91 to 977.63, *p* = 0.005). Clearly, however, if Leaders were still learning in the L-R environment, this was true for about two thirds of them, not all of them, and some had similarly straight Return paths to those of Leaders in the L-P environment. Indeed, mean path tortuosity was considerably higher for Leaders in the L-R than in the L-P environment but the variation was more than three times greater and the means were significantly different at the 10% significance level but not at the 5% significance level (Fig. 4; Table 1).

The number of Followers with Return paths containing loops was the same for the L-R and the L-P environments (14 out of 17 in both L-R and L-P, Ants 16–18, 21–31 in L-R, Ants 48, 50, 52–62, 64 in L-P, Fig. S5). Even though the mean path tortuosity for returning Followers was lower in the L-R than in the L-P environment, the difference was not statistically significant (Fig. 4; Table 1).

In the comparisons between Leaders and Followers within and between the two environments, there was a significant difference between the path tortuosity of the Leaders in the L-P environment and the Followers in each of the L-P and L-R environments (Fig. 4; Table 1). This is in agreement with our expectation that Leaders in the L-P environment and Followers in either the L-P or L-R environment should have the lowest and the highest value of path tortuosity, respectively. The absence of a significant difference in path tortuosity between Leaders in the L-R environment and Followers in either environment (Fig. 4; Table 1), however, is not in agreement with our expectation that since leaders are more knowledgeable about the route in both environments, they should have lower path tortuosity and higher mean speed than followers irrespective of the environment.

**Fig. 5:**
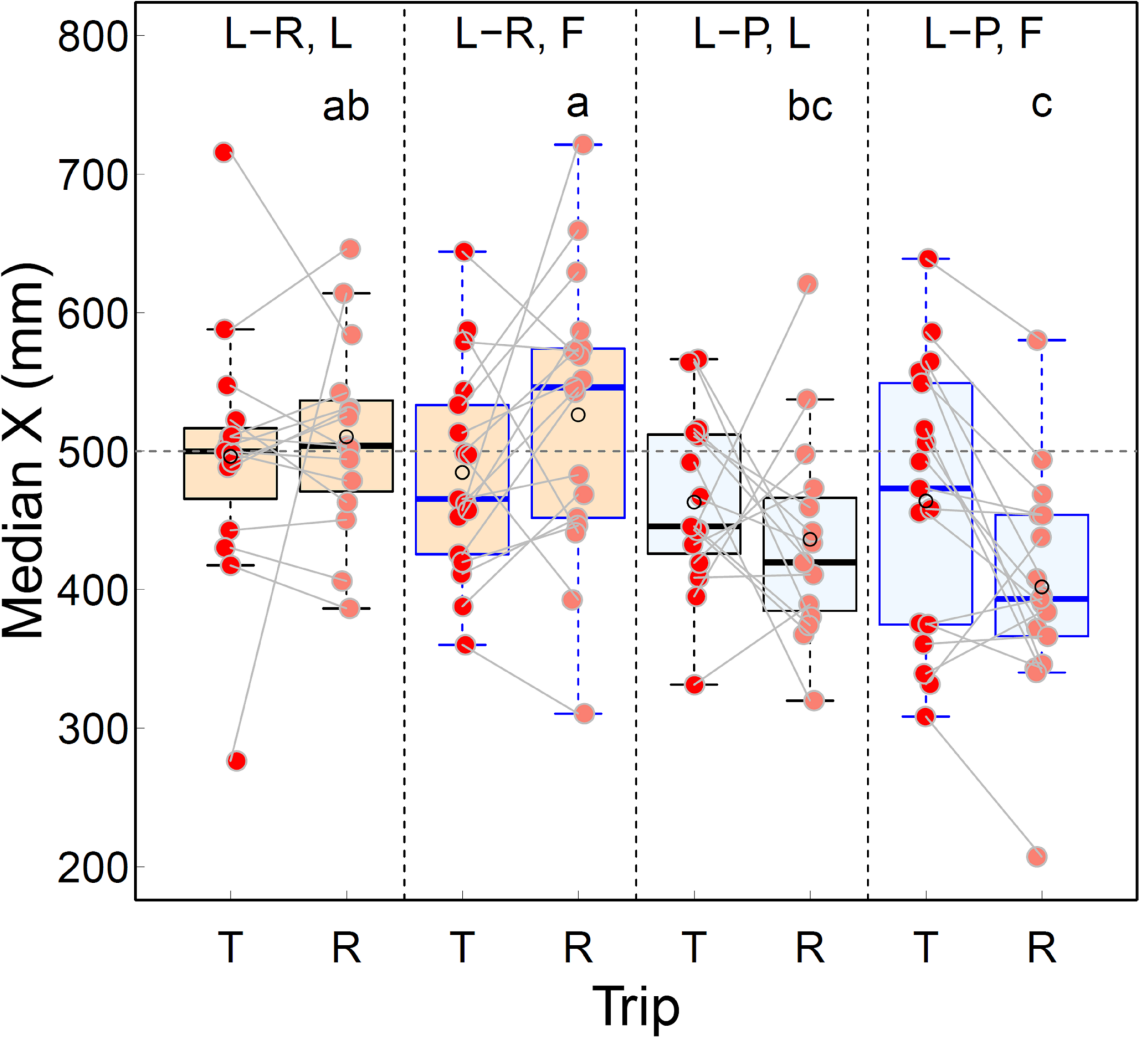
The effect of Treatment (Landmark-rich or Landmark-poor), Role (Leader or Follower) and Trip (Tandem or Return) on the median X (mm) for the path. Everything else is as in the caption for Fig. 4, except that the post-hoc pair-wise comparisons between means for the Return trips, some of which were significant at the 5% significance level, are in Table 2 and the differences between means for the Tandem trips, all of which were not significant, are in Table S7; the horizontal grey interrupted line represents the axis between the entrances of the ON and the NN

Intriguingly, the paths of both Leaders and Followers in the L-P Treatment were biased to the right side on the Return trip, namely most of them tended to be in the half of the arena from 0 to 500 cm along the x axis (Fig. 3C, D), where X = 500 cm represents the line between the entrances of the ON and the NN. Indeed, the Interquartile Range (IQR) for the distribution of median X value (mm) of the path did not include 500 cm for the Return trip of either Leaders or Followers in the L-P Treatment. In fact, only one Follower ant and two Leader ants had a median X value greater than 500 cm on the Return trip in the L-P Treatment (Fig. 5, L-PLR and L-PFR). By contrast all other paths, on both the Tandem and Return trip, were approximately symmetrically distributed around the middle of the arena’s x-axis at 500 cm (Figs. 2A-D and 3A, B). The visible bias to the right of the paths on the Return trip in the L-P treatment was reflected in the statistical significance of three out of the four possible pair-wise comparisons between the median X value of the Return path for Leaders and Followers across the two treatments (Fig. 5; Table 2).

**Table 1:** Post-hoc pair-wise comparisons between means in the best GLMMs for path tortuosity and mean speed (mm/s) on the return trip

Compared means	Path tort. Ho: Diff.=0	SE	Z	*P*	Mean speed (mm/s) Ho: Diff.=0	SE	Z	*P*
L-RLR - L-RFR	-0.055	0.063	-0.885	0.964	0.013	0.624	0.020	1.000
L-RLR - L-PLR	*0.169*	*0.065*	*2.624*	*0.084*	-1.120	0.644	-1.739	0.514
L-RLR - L-PFR	-0.108	0.063	-1.726	0.521	0.970	0.624	1.553	0.646
L-RFR - L-PLR	**0.225**	**0.063**	**3.590**	**0.004**	-1.132	0.624	-1.813	0.462
L-RFR - L-PFR	-0.053	0.061	-0.869	0.967	0.957	0.605	1.583	0.625
L-PLR - L-PFR	**-0.277**	**0.063**	**-4.431**	**< 0.001**	**2.089**	**0.624**	**3.346**	**0.009**

Each difference between compared means is based on the product between the model coefficients in Tables S2A (path tortuosity) and S4A (mean speed), and the multipliers in Tables S2D-E. Ho stands for null hypothesis and Diff. stands for the difference between the respective means. Cells highlighted in bold indicate a significant difference at the 5% significance level; cells highlighted in italics indicate a significant difference at the 10% significance level.

**Table 2:** Post-hoc pair-wise comparisons between means in the best GLMM for median X (mm) on the return trip

Compared means	Median X (mm) Ho: Diff.=0	SE	Z	*P*
L-RLR - L-RFR	510.40–526.25 = -15.85	29.832	-0.531	1.000
L-RLR - L-PLR	510.40–436.34 = 74.06	30.750	2.409	0.142
L-RLR - L-PFR	**510.40–402.00 = 108.40**	**29.832**	**3.634**	**0.003**
L-RFR - L-PLR	**526.25–436.34 = 89.91**	**29.832**	**3.014**	**0.027**
L-RFR - L-PFR	**526.25–402.00 = 124.25**	**28.884**	**4.302**	**< 0.001**
L-PLR - L-PFR	436.34–402.00 = 34.34	29.832	1.151	0.873

Each difference between compared means is based on the product between the model coefficients in Table S3A and the same multipliers as those in Tables S2D-E. Ho stands for null hypothesis and Diff. stands for the difference between the respective means. The values of the means are given to allow comparison with the axis between the ON and the NN at x = 500 mm. Cells highlighted in bold indicate a significant difference at the 5% significance level.

### Mean speed

**Fig. 6:**
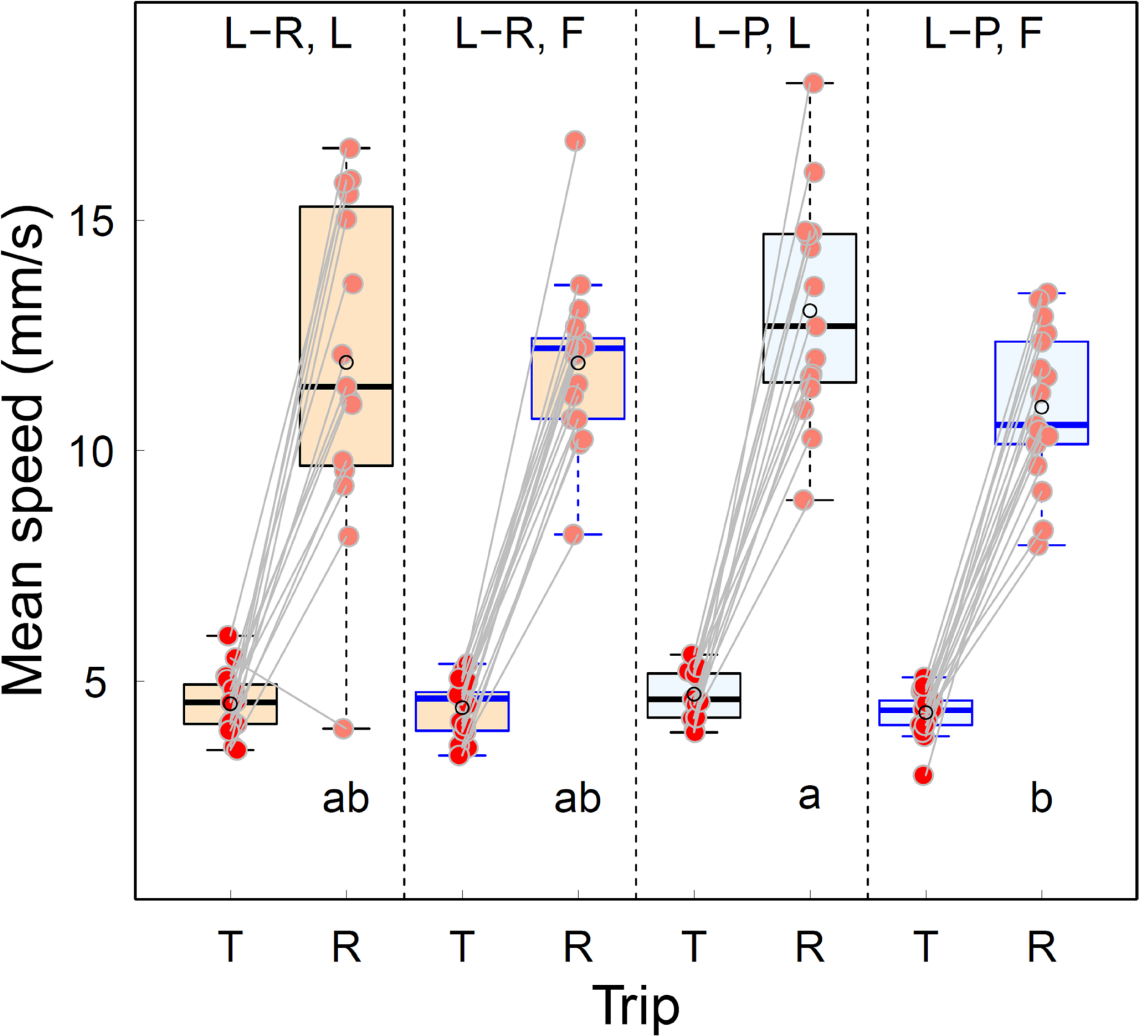
The effect of Treatment (Landmark-rich or Landmark-poor), Role (Leader or Follower) and Trip (Tandem or Return) on mean speed. Everything else is as in the caption for Fig. 4. Ant 15, the only individual to have a lower mean speed on the Return than on the Tandem trip (L-R, L, mean speed for the T trip is greater than its paired value for the R trip), is a notable outlier but we found that the removal of its data points did not have a significant effect (Fig. S3)

The mean speed of individuals tended to be higher on the Return trip than on the Tandem trip for both Roles (L and F) in both Treatments (L-R and L-P, paired values for individual ants in Fig. 6, only Ant 15 makes an exception). There were no significant differences in Tandem mean speed in any of the pair-wise comparisons between Treatment × Role combinations (Fig. 6, Table S6).

The order of the four mean speeds on the Return trip were in agreement with our expectations based on Leaders continuing to learn navigational information in the L-R environment and Followers having more to learn on their way back to the ON in both environments. Leaders tended to move more quickly than Followers in either environment (Fig. 6; Table 1). However, the only significant difference was between the means for Leaders and Followers in the L-P environment (Fig. 6; Table 1). This is in agreement with our expectation that the difference between mean speed for Leaders in the L-P environment and Followers in either the L-P or the L-R environment would be the largest.

### Distance between tandem and return paths

We found no evidence that either Leaders or Followers followed the Tandem paths on their Return trip in either treatment. Even the shortest mean distance between the two paths for an individual was longer than 20 mm (Fig. S6, see also Fig. S5). This is more than 10 times the typical distance between each ant’s antennae. We also found no evidence of a difference in the ants’ distances between Tandem and Return paths according to whether they were a Leader or a Follower and whether they were in the L-R or the L-P environment. All of the six possible post-hoc pair-wise comparisons between means in the best GLM for distance between Tandem and Return paths had absolute Z values between 0.107 and 1.652 and respective p-values between 1.000 and 0.349 (Table S8).

## Discussion

We expected that if Leaders were still learning navigational information on the Return trip (when ants make their own way back to the ON) in the L-R environment, their paths would contain loops similar to those in the paths of returning Followers. We found that the odds of loops being present in the Return path were significantly higher for Leaders in the L-R than in the L-P environment and that the values for mean tortuosity and mean speed for Leaders and Followers in the two environments were ordered in agreement with our expectation. There were, however, two unexpected results.

First, the Return paths of only about two thirds of the Leaders in the L-R environment contained loops. Although, this was significantly more than for Leaders in the L-P environment, it suggests a heterogeneity in behaviour: some, not necessarily all, Leaders learn on their way back to the ON when rich navigational information is available in the environment. Such heterogeneity almost certainly contributed to the lack of statistical significance in some of the comparisons between the means for tortuosity and speed on the Return trip for Leaders and Followers in the two environments. In this sense, our results might appear weaker than expected. However, our experimental procedure afforded equal opportunities for potential tandem Leaders to explore the arena, discover and evaluate the NN, and make the decision to teach the route to it. We did not measure the duration of the experience future tandem leaders and followers had in the arena and it is possible that the more experienced leaders made fewer loops and had less tortuous return paths. For example, studies with desert ants have found that with experience, ants look less and strike straighter return paths in the context of dumping and foraging (Deeti and Cheng 2024; Freas and Cheng 2025). We suggest that in the future an experiment is designed specifically to test whether ants with longer prior experience of the arena in a landmark-rich environment make fewer loops and strike straighter paths on their return journeys in the context of tandem running during ant colony emigration. If such an association does exist for leaders, it would still suggest that leaders with shorter prior experience of the arena are learning on their return trips in an environment rich in navigational information. After all, the length of prior experience of the arena in the L-P environment did not seem to have an effect on the return trips of Leaders probably because there was not so much to learn. Therefore, the heterogeneity in the presence of path loops as evidence of continued learning is intriguing. It suggests that different ants with the ability to lead a tandem run respond differently to the availability of rich navigational information. One possibility is that some former tandem leaders make loops in their return paths because they learn more when there is more to learn and that makes them better recruiters. Another possibility is that tandem leaders that have a short prior experience of the arena, because they happen to find the new nest quickly, save time by learning more about a rich navigational environment on their return trip instead of during prior exploration. The result in either case is that tandem leaders become specialised in that task by taking it away from nestmates in a self-reinforcing process. Indeed, this might be one of the mechanisms for the formation of leader oligarchies in tandem running (Richardson et al. 2018).

Second, the Return paths of both Leaders and Followers were biased to the right side on the way back to the ON in the environment poor in landmarks. Thus their location along the line between the two nests was significantly different from that of the Return paths of Leaders and Followers in the environment rich in navigational information. This suggests ants use different navigational mechanisms in the two environments.

An alternative explanation of our results is that there is no continued leaning by, at least some of, the Leaders on their Return trips in the L-R environment. Instead, they make loops because this is just what is needed in an environment rich in navigational information. Such an alternative seems improbable for two main reasons. First, the emigration has to proceed as quickly as possible because the colony is vulnerable to predation and the elements. Unless there is a benefit such as learning the route better, a more tortuous path would make the journey longer. Therefore, Leaders in the environment richer in navigational information cannot afford to have more tortuous paths than possible in the environment with less navigational information. Second, the very presence of Leaders in L-R with straight Return paths similar to those of Leaders in L-P demonstrates that loops are not needed.

We found no evidence that Leaders or Followers follow the Tandem path on their Return trip in either treatment but the bias to the right side on the way back to the ON in the paths of both Leaders and Followers in the L-P treatment could be a clue to the way they orientate in a poor navigational environment. Workers in this species have a behavioural lateralization towards the right (Basari et al. 2014a; Hunt et al. 2018). The arena wall, folds in the muslin covering the experimental set-up and the strip light over which it was suspended could have given directional cues.

By contrast to the Return trip, the difference in the available navigational information in the L-R and L-P treatments did not affect the mean speed or path tortuosity of ants during the Tandem trip. This is probably because tandem running represents a teaching opportunity (Franks and Richardson 2006): leaders lead tandem runs along a twisting path (Franklin et al. 2012) and followers loop around during pauses to learn landmarks (Franks and Richardson 2006). Hence any differences in mean speed and tortuosity between the two treatments would be comparatively small. Furthermore, given that they are in a tandem, any differences between Leaders and Followers within each of the two treatments would be even smaller. All this suggests that Leaders move slowly on a tortuous path during the tandem run to the new nest predominantly to give a learning opportunity to their followers rather than to themselves. This result is consistent with the demonstration of teaching in ants by Franks and Richardson (2006) because it shows once again that in the context of tandem running ants, the leader ant modifies its behaviour in the presence of a naïve follower, thus meeting one of the main criteria for teaching in animals (Caro and Hauser 1992; Richardson et al. 2007).

From all our results above, the most surprising is the evidence that even knowledgeable tandem leaders take advantage of the additional information available in the landmark-rich environment on their return trip from the new nest back to the old nest. They do this even though they must have collected landmark information before their initial decision to lead a tandem run to the new nest. Our evidence is that their paths are more tortuous than they could be because in the landmark-poor environment the leaders’ paths are noticeably straighter. Previous studies of the same ants (Franklin et al. 2012) and pigeons (Guilford and Biro 2014) have suggested that the subsequent journey paths of individuals that are knowledgeable about the route are less tortuous compared to those of less knowledgeable individuals. So, what are former leaders doing on their return paths home? At least some of them appear to be performing looping searches to learn even more ways to find the way back home. This is similar to follower ants performing looping searches during the tandem run, most likely in order to learn the way home while proceeding ahead to the new nest (Franks and Richardson 2006). When foraging, outbound ants turn around and retrace their steps and thus they acquire views that help to guide them on their inbound journey (Collett and Graham 2004). The outbound and inbound view of the route may be different (Collett et al. 2003) but by doing a looping search, ants during tandem-run and return trips may provide themselves with an opportunity to learn both views in the context of tandem running in a similar way to ants retracing their steps during foraging.

Route landmarks, such as the computer behind the old nest, available in the landmark-rich treatment of the present study, could act as a beacon landmark (Graham and Collett 2002) and the arena wall, available in both treatments, could provide another cue to the navigating ants (Pratt et al. 2001), which might have been particularly important in the landmark-poor treatment of the present study. As previous studies have suggested, route memory cannot be established instantaneously but develops gradually over time (Collett et al. 1993). Hence ants may learn bit by bit as they walk along the path, becoming more familiar with landmarks and the overall panoramic view as they use the same route repeatedly (Baddeley et al. 2011). Our results suggest that such additional learning tends to take place in environments with richer navigational information.

Visual cues are crucial for navigation by tandem-running ants. In general, ants that are knowledgeable about the route tend to navigate by relying on visual landmarks while naïve ants rely more on chemical cues (Harrison et al. 1989 but see also Schultheiss et al. 2015). However, even though the follower ant can probably use a chemical trail laid by the tandem leader during a tandem run (Basari et al. 2014b) to assist in taking a direct route back to the old nest, it seems that these ants only use trails as a general orientation cue (Pratt et al. 2001). Indeed, the results of Bowens et al. (2013, 2015) suggest that *T. curvispinosus* ants use chemical signals for marking familiar territory rather than for orientation. Therefore, *Temnothorax* ants do not follow the exact trail as other species of trail laying ants do (for example, as in the army ants, *Eciton burchellii*, Witte and Maschwitz 2002; Couzin and Franks 2003). Moreover, as corroborated by the present study, the return paths of *T. albipennis* ants do not overlay their tandem paths (Franks et al. 2010; Basari et al. 2014a; Sasaki et al. 2020), suggesting that they may rely more on visual cues than chemical trails when returning to the old nest.

Other than using solely visual cues, ants are known to use path integration to help them navigate (Wehner and Srinivasan 2003) and they may also use both visual cues and path integration to help them navigate better (Narendra 2007; Wystrach and Graham 2012; Zeil et al. 2014; Graham and Mangan 2015). However, in using path integration, the animal needs to update its home vector continuously. For most animals, when they complete their journey, they have a simple task of picking up a prey item before heading home. During nest relocation, ants make an outward journey to the new nest and then need to evaluate the new nest site, a process that may involve a separate set of calculations (Mallon and Franks 2000), before eventually heading back to the old nest site. Both the leader and the follower in a tandem run spend some time in the new nest after they arrive there. The tandem follower needs to make her own assessment of the inside of the new nest before heading back home. It is yet unknown what happens to the path integration mechanism during the movement involved in such evaluations. Does it continue to update the home vector and thus accumulate errors (Heinze et al. 2018; Collett 2019; Freire et al. 2023) or does it stop updating on entry in the new nest, which would then require a retrieval of that value when the ant exits? Hence, in an emigration process, the calculation and use of a home vector might be impaired compared to a simple foraging task.

Overall, the results of the present study strongly suggest that tandem running helps not only the follower ant to learn the route to a new resource but also presents an opportunity for the leader to update navigational information, and hence, improve her knowledge of the route between the old and the new nests. Our results strongly support and expand on the previous findings of Franklin et al. (2012) that with tandem-running experience (both as the leader and the follower), returning ants can improve their navigation to become more efficient. The quicker the members of former tandem runs return to the old nest, the faster they can recruit others and the faster the emigration will proceed, thus minimizing exposure of the colony to any predators and inclement environmental conditions. Such an updating mechanism may also be one of the self-reinforcing processes that underpin the formation of leader oligarchies in tandem running and facilitate the control such oligarchies exert on the flow of information during house hunting in *T. albipennis* ants (Richardson et al. 2018). Last but not least, our study highlights that the reinforcement of navigational information is of tremendous importance for tandem leaders as well as for tandem followers.

## Electronic supplementary material

Below is the link to the electronic supplementary material.

Supplementary Material 1

Supplementary Material 2

Supplementary Material 3

Supplementary Material 4

Supplementary Material 5

Supplementary Material 6

## Data Availability

Data is provided within the manuscript or supplementary information files.
