# Supplementary material for: Differential response by tandem leaders and followers to landmark-rich and landmark-poor environments
Source: Anim Cogn. 2025 May 19;28(1):40. doi: 10.1007/s10071-025-01958-x (PMC12089173; doi:10.1007/s10071-025-01958-x)
Supplement: Supplementary file 6 — Supplementary Material 6 [file 10071_2025_1958_MOESM6_ESM.docx]

**Supplementary Information**

**Journal name: Animal Cognition**

# Differential Response by Tandem Leaders and Followers to Landmark-Rich and Landmark-Poor Environments

Norasmah Basari, Ana B. Sendova- Franks^*^, Alan Worley and Nigel R. Franks

^*^Corresponding author:

Address: School of Biological Sciences, Bristol Life Sciences Building, University of Bristol, 24 Tyndall Avenue, Bristol, BS8 1TQ, UK

E-mail: [ana.sendova-franks@bristol.ac.uk](mailto:ana.sendova-franks@bristol.ac.uk)

**Table S1** The number of ants with a Tandem and Return trip from each Colony classified according to Role (Leader or Follower) and Treatment (L-R, Landmark-rich or L-P, Landmark-poor).

|  | Leader | | Follower | |
| --- | --- | --- | --- | --- |
| Colony | L-R | L-P | L-R | L-P |
| 1 | 0 | 2 | 0 | 3 |
| 2 | 1 | 0 | 1 | 1 |
| 3 | 3 | 0 | 2 | 0 |
| 4 | 2 | 2 | 2 | 2 |
| 5 | 1 | 1 | 2 | 1 |
| 6 | 0 | 4 | 3 | 3 |
| 7 | 3 | 0 | 3 | 0 |
| 8 | 1 | 3 | 1 | 3 |
| 9 | 1 | 1 | 1 | 1 |
| 10 | 2 | 0 | 1 | 0 |
| 11 | 1 | 0 | 1 | 0 |
| 12 | 0 | 2 | 0 | 3 |
| Total | 15 | 15 | 17 | 17 |

**Table S2A** Best model for path tortuosity, modt1.2

| Fixed effects: | Estimate | Std. Error | df | t value | Pr(>\|t\|) |
| --- | --- | --- | --- | --- | --- |
| (Intercept) | 0.457000 | 0.042810 | 117.9754 | 10.675 | < 2e-16 |
| TreatmentL-R | -0.052588 | 0.060542 | 117.9754 | -0.869 | 0.38682 |
| RoleL | -0.277067 | 0.062528 | 117.9754 | -4.431 | 2.11E-05 |
| TripT | 0.115706 | 0.056437 | 60.0000 | 2.050 | 0.04473 |
| TreatmentL-R:RoleL | 0.221722 | 0.088427 | 117.9754 | 2.507 | 0.01352 |
| TreatmentL-R:TripT | -0.007118 | 0.079815 | 60.0000 | -0.089 | 0.92924 |
| RoleL:TripT | 0.238427 | 0.082432 | 60.0000 | 2.892 | 0.00532 |
| TreatmentL-R:RoleL:TripT | -0.168216 | 0.116577 | 60.0000 | -1.443 | 0.15423 |

The best model was selected using the AIC-minimisation criterion. For more details of the alternative models, see the subsection Statistical analysis in the main paper. Treatment could have one of two values: Landmark-rich (L-R) or Landmark-poor (L-P), Role could be either Leader (L) or Follower (F) and Trip could be either Tandem (T) or Return (R). The category not visible in the respective model coefficient name is the reference category.

**Table S2B** Path tortuosity, modt1.2, Random effects

| Groups Name | Variance | Std. Dev. |
| --- | --- | --- |
| ID (Intercept) | 0.004081 | 0.06389 |
| Residual | 0.027074 | 0.16454 |

Number of obs: 128, groups: ID, 64, ID stands for Ant ID

**Table S2C** Path Tortuosity, modt1.2, Scaled residuals

| Min | 1Q | Median | 3Q | Max |
| --- | --- | --- | --- | --- |
| -1.70938 | -0.71675 | -0.09569 | 0.69370 | 1.97470 |

**Table S2D** Multipliers for the model coefficients for Path tortuosity in column ‘Estimate’, Table S2A, needed to obtain the model equation for each combination of the three factors by two levels each

| Factor combination | Multipliers for the eight model coefficients |
| --- | --- |
| L-RLT | 1, 1, 1, 1, 1, 1, 1, 1 |
| L-RLR | 1, 1, 1, 0, 1, 0, 0, 0 |
| L-RFT | 1, 1, 0, 1, 0, 1, 0, 0 |
| L-RFR | 1, 1, 0, 0, 0, 0, 0, 0 |
| L-PLT | 1, 0, 1, 1, 0, 0, 1, 0 |
| L-PLR | 1, 0, 1, 0, 0, 0, 0, 0 |
| L-PFT | 1, 0, 0, 1, 0, 0, 0, 0 |
| L-PFR | 1, 0, 0, 0, 0, 0, 0, 0 |

The three factors are the following. Treatment: Landmark-rich (L-R, coded as 1) or Landmark-poor (L-P, coded as 0, reference level), Role: Leader (L, 1) or Follower (F, 0) and Trip: Tandem (T, 1) or Return (R, 0). The equation for a given combination of factor levels is obtained by calculating the product of the model coefficients in Table S2A and the respective eight multipliers in the present table. For example, for factor combination L-RLT, all eight multipliers are 1s and hence all the model coefficients for it are as in Table S2A. At the other end, for factor combination L-PFR, only the first multiplier is 1 and hence its only model coefficient is the intercept in Table S2A.

**Table S2E** Multipliers for the model coefficients for Path tortuosity in column ‘Estimate’, Table S2A, needed to obtain the difference between the means for a priori chosen comparisons between combinations of the three factors with two levels each

| A priori chosen comparisons between factor combinations | Multipliers for the eight model coefficients |
| --- | --- |
| L-RLR - L-RFR | 0, 0, 1, 0, 1, 0, 0, 0 |
| L-RLR - L-PLR | 0, 1, 0, 0, 1, 0, 0, 0 |
| L-RLR - L-PFR | 0, 1, 1, 0, 1, 0, 0, 0 |
| L-RFR - L-PLR | 0, 1, -1, 0, 0, 0, 0, 0 |
| L-RFR - L-PFR | 0, 1, 0, 0, 0, 0, 0, 0 |
| L-PLR - L-PFR | 0, 0, 1, 0, 0, 0, 0, 0 |
| L-RLT - L-RFT | 0, 0, 1, 0, 1, 0, 1, 1 |
| L-RLT - L-PLT | 0, 1, 0, 0, 1, 1, 0, 1 |
| L-RLT - L-PFT | 0, 1, 1, 0, 1, 1, 1, 1 |
| L-RFT - L-PLT | 0, 1, -1, 0, 0, 1, -1,0 |
| L-RFT - L-PFT | 0, 1, 0, 0, 0, 1, 0, 0 |
| L-PLT - L-PFT | 0, 0, 1, 0, 0, 0, 1, 0 |

The a priori chosen comparisons were all six possible factor-level combinations for the Return journey, on the one hand, and all six possible factor-level combinations for the Tandem journey, on the other (see main text for justification). For example, for the difference between means L-RLR - L-RFR, the multipliers represent the differences between the respective multipliers for L-RLR and L-RFR in Table S2D. Only the third and fifth of these multipliers (first row above) are 1s. Therefore, we need to use only the third and fifth coefficients from the Tortuosity model in Table S2a above. This gives -0.277067+0.221722 = -0.055345. Indeed, -0.055 is the difference between these means in Table 1.

**Table S3A** Best model for median X (mm), modMx1.2

| Fixed effects: | Estimate | Std. Error | df | t value | Pr(>\|t\|) |
| --- | --- | --- | --- | --- | --- |
| (Intercept) | 402.00 | 20.42 | 108.87 | 19.682 | < 2e-16 |
| TreatmentL-R | 124.25 | 28.88 | 108.87 | 4.302 | 3.71E-05 |
| RoleL | 34.34 | 29.83 | 108.87 | 1.151 | 0.2522 |
| TripT | 62.05 | 23.82 | 60.00 | 2.604 | 0.0116 |
| TreatmentL-R:RoleL | -50.19 | 42.19 | 108.87 | -1.190 | 0.2368 |
| TreatmentL-R:TripT | -103.66 | 33.69 | 60.00 | -3.077 | 0.0032 |
| RoleL:TripT | -35.17 | 34.80 | 60.00 | -1.011 | 0.3162 |
| TreatmentL-R:RoleL:TripT | 62.65 | 49.21 | 60.00 | 1.273 | 0.2079 |

The best model was selected using the AIC-minimisation criterion. For more details of the alternative models, see the subsection Statistical analysis in the main paper. All other details are as in the caption for Table S2A.

**Table S3B** Median X (mm), modMx1.2, Random effects

| Groups Name | Variance | Std. Dev. |
| --- | --- | --- |
| ID (Intercept) | 2267 | 47.61 |
| Residual | 4825 | 69.46 |

Number of obs: 128, groups: ID, 64, ID stands for Ant ID

**Table S3C** Median X (mm), modMx1.2, Scaled residuals

| Min | 1Q | Median | 3Q | Max |
| --- | --- | --- | --- | --- |
| -2.76076 | -0.54113 | -0.03543 | 0.52910 | 2.22402 |

**Table S4A** Best model for mean speed (mm/s), mods1.2

| Fixed effects: | Estimate | Std. Error | df | t value | Pr(>\|t\|) |
| --- | --- | --- | --- | --- | --- |
| (Intercept) | 10.9448 | 0.4275 | 119.623 | 25.602 | < 2e-16 |
| TreatmentL-R | 0.9573 | 0.6046 | 119.623 | 1.583 | 0.1160 |
| RoleL | 2.0893 | 0.6244 | 119.623 | 3.346 | 1.10E-03 |
| TripT | -6.6171 | 0.5874 | 60.000 | -11.266 | < 2e-16 |
| TreatmentL-R:RoleL | -2.0768 | 0.8830 | 119.623 | -2.352 | 0.0203 |
| TreatmentL-R:TripT | -0.8516 | 0.8307 | 60.000 | -1.025 | 0.3094 |
| RoleL:TripT | -1.6900 | 0.8579 | 60.000 | -1.970 | 0.0535 |
| TreatmentL-R:RoleL:TripT | 1.7621 | 1.2132 | 60.000 | 1.452 | 0.1516 |

The best model was selected using the AIC-minimisation criterion. For more details of the alternative models, see the subsection Statistical analysis in the main paper. All other details are as in the caption for Table S2A.

**Table S4B** Mean speed (mm/s), mods1.2, Random effects

| Groups Name | Variance | Std. Dev. |
| --- | --- | --- |
| ID (Intercept) | 0.1744 | 0.4176 |
| Residual | 2.9324 | 1.7124 |

Number of obs: 128, groups: ID, 64, ID stands for Ant ID

**Table S4C** Mean speed (mm/s), mods1.2, Scaled residuals

| Min | 1Q | Median | 3Q | Max |
| --- | --- | --- | --- | --- |
| -4.4192 | -0.3753 | 0.0368 | 0.3138 | 2.7524 |

**Table S5A** Best model for distance between Tandem and Return path (mm), modp0

| Coefficients: | Estimate | Std. Error | t value | Pr(>\|t\|) |
| --- | --- | --- | --- | --- |
| (Intercept) | 1.84140 | 0.05687 | 32.380 | < 2e-16 |
| TreatmentL-R | 0.07094 | 0.08042 | 0.882 | 0.3813 |
| RoleL | 0.06207 | 0.0831 | 0.747 | 0.4578 |
| TreatmentL-R:RoleL | -0.19932 | 0.11747 | -1.697 | 0.0949 |

The best model was selected using the AIC-minimisation criterion. For more details of the alternative models, see the subsection Statistical analysis in the main paper. All other details are as in the caption for Table S2A, except that the predictor Trip is absent for obvious reasons.

**Table S5B** T to R Distance (mm), modp0, Scaled residuals

| Min | 1Q | Median | 3Q | Max |
| --- | --- | --- | --- | --- |
| -2.0523 | -0.7336 | 0.1077 | 0.7102 | 3.0138 |

**Table S5C** Multipliers for the model coefficients for Distance between Tandem and Return path (mm), in column ‘Estimate’, Table S5a, needed to obtain the equation for each combination of the two factors by two levels each

| Factor combinations | Multipliers for the four model coefficients |
| --- | --- |
| L-RL | 1, 1, 1, 1 |
| L-RF | 1, 1, 0, 0 |
| L-PL | 1, 0, 1, 0 |
| L-PF | 1, 0, 0, 0 |

The two factors are the following. Treatment: Landmark-rich (L-R, coded as 1) or Landmark-poor (L-P, coded as 0, reference level) and Role: Leader (L, 1) or Follower (F, 0). The equation for a given combination of factor levels is obtained by calculating the product of the model coefficients in Table S5a and the respective four multipliers in the present table. For example, for factor combination L-RL, all four multipliers are 1s and hence all the model coefficients for it are as in Table S5A. At the other end, for factor combination L-PF, only the first multiplier is 1 and hence its only model coefficient is the intercept in Table S5A.

**Table S5D** Multipliers for the model coefficients for Distance between Tandem and Return path (mm) in column ‘Estimate’, Table S5A, needed to obtain the difference between the means for a priori chosen comparisons between combinations of the two factors with two levels each

| All possible comparisons between factor combinations | Multipliers for the four model coefficients |
| --- | --- |
| L-RL - L-RF | 0, 0, 1, 1 |
| L-RL - L-PL | 0, 1, 0, 1 |
| L-RL - L-PF | 0, 1, 1, 1 |
| L-RF - L-PL | 0, 1, -1, 0 |
| L-RF - L-PF | 0, 1, 0, 0 |
| L-PL - L-PF | 0, 0, 1, 0 |

The a priori chosen comparisons were all six possible factor-level combinations. For example, for the difference between means L-RL - L-RF, the multipliers represent the differences between the respective multipliers for L-RL and L-RF in Table S5C. Only the third and fourth of these multipliers (first row above) are 1s. Therefore, we need to use only the third and fourth coefficients from the model for the distance between Tandem and Return paths in Table S5A above. This gives 0.06207 - 0.19932 = -0.13725. Indeed, -0.1373 is the difference between these means in Table S8.

**Table S6** Post-hoc pair-wise comparisons between means in the best GLMMs for path tortuosity and mean speed (mm/s) on the Tandem trip

| Compared  means | Path tort.  Ho: Diff.=0 | SE | Z | *P* | Mean speed (mm/s)  Ho: Diff.=0 | SE | Z | *P* |
| --- | --- | --- | --- | --- | --- | --- | --- | --- |
| L-RLT - L-RFT | 0.015 | 0.063 | 0.238 | 1.000 | 0.085 | 0.624 | 0.135 | 1.000 |
| L-RLT - L-PLT | -0.006 | 0.065 | -0.096 | 1.000 | -0.209 | 0.644 | -0.325 | 1.000 |
| L-RLT - L-PFT | -0.045 | 0.063 | -0.717 | 0.988 | 0.190 | 0.624 | 0.305 | 1.000 |
| L-RFT - L-PLT | -0.021 | 0.063 | -0.337 | 1.000 | -0.294 | 0.624 | -0.470 | 0.999 |
| L-RFT - L-PFT | -0.060 | 0.061 | -0.986 | 0.940 | 0.106 | 0.605 | 0.175 | 1.000 |
| L-PLT - L-PFT | -0.039 | 0.063 | -0.618 | 0.994 | 0.399 | 0.624 | 0.639 | 0.993 |

All details are as for Table 1 in the main text.

**Table S7** Post-hoc pair-wise comparisons between means in the best GLMM for median X (mm) on the Tandem trip

| Compared  means | Median X (mm)  Ho: Diff.=0 | SE | Z | *P* |
| --- | --- | --- | --- | --- |
| L-RLT - L-RFT | 496.27 – 484.63 = 11.64 | 29.832 | 0.390 | 1.000 |
| L-RLT - L-PLT | 496.27 – 463.21 = 33.06 | 30.750 | 1.075 | 0.905 |
| L-RLT - L-PFT | 496.27 – 464.05 = 32.22 | 29.832 | 1.080 | 0.903 |
| L-RFT - L-PLT | 484.63 – 463.21 = 21.42 | 29.832 | 0.718 | 0.986 |
| L-RFT - L-PFT | 484.63 – 464.05 = 20.58 | 28.884 | 0.713 | 0.987 |
| L-PLT - L-PFT | 463.21 – 464.05 = -0.84 | 29.832 | -0.028 | 1.000 |

All details are as for Table 2 in the main text.

**Table S8** Post-hoc pair-wise comparisons between means in the best GLM for distance between Tandem and Return paths (mm)

| Compared  means | T-R path distance (mm)  Ho: Diff.=0 | SE | Z | *P* |
| --- | --- | --- | --- | --- |
| L-RL - L-RF | -0.1373 | 0.08306 | -1.652 | 0.349 |
| L-RL - L-PL | -0.1284 | 0.08562 | -1.499 | 0.438 |
| L-RL - L-PF | -0.0663 | 0.08306 | -0.798 | 0.855 |
| L-RF - L-PL | 0.0089 | 0.08310 | 0.107 | 1.000 |
| L-RF - L-PF | 0.0709 | 0.08042 | 0.882 | 0.814 |
| L-PL - L-PF | 0.0621 | 0.08306 | 0.747 | 0.878 |

Each difference between compared means is based on the product between the model coefficients and the multipliers in Tables S5C-D. Ho stands for null hypothesis and Diff. stands for the difference between the respective means.

**Fig. S1** A normal Q-Q plot of the residuals for the best model for tortuosity; the black line represents the expected position of the points in the absence of any deviations from the Normal distribution; black empty circles represent the Landmark-rich (L-R) treatment, red empty circles represent the Landmark-poor (L-P) treatment. There is some deviation from expectation in the tails and some evidence of platykurtosis, namely a flatter distribution with thinner tails than the Normal distribution: the points in the right-hand tail are below the line and those in the left-hand tail are above the line.

**Fig. S2** A normal Q-Q plot of the residuals for the best model for median path x-value (mm); all the details are as in the caption for Fig. S1. Most of the points are wrapped around the line of expectation and hence there is no evidence of notable deviations from the Normal distribution.

**Fig. S3** A normal Q-Q plot of the residuals for the best model for mean speed (mm/s); all the details are as in the caption for Fig. S1. There is some deviation from expectation in the tails and some evidence of leptokurtosis, namely a more pointed distribution with fatter tails than the Normal distribution: the points in the right-hand tail are above the line and those in the left-hand tail are below the line. The point in the bottom left corner with a residual of about -8 is for Ant 15, the only individual to have a lower mean speed on the Return trip than on the Tandem trip. The removal of the paired values for the T and R trips for Ant 15 under Treatment L-R and Role L improved the model fit (range of scaled residuals: -2.50 to 2.98, compared to values for model mods1.2 in Table S4c, Shapiro-Wilk Normality test for scaled residuals: W=0.9556, p=3.977*10^-4^). However, the deviations from normality remained in both tails of the distribution of the residuals.

**Fig. S4** A normal Q-Q plot of the residuals for the best model for distance between T and R path (mm); all the details are as in the caption for Fig. S1. Most of the points are wrapped around the line of expectation and hence there is no evidence of notable deviations from the Normal distribution.


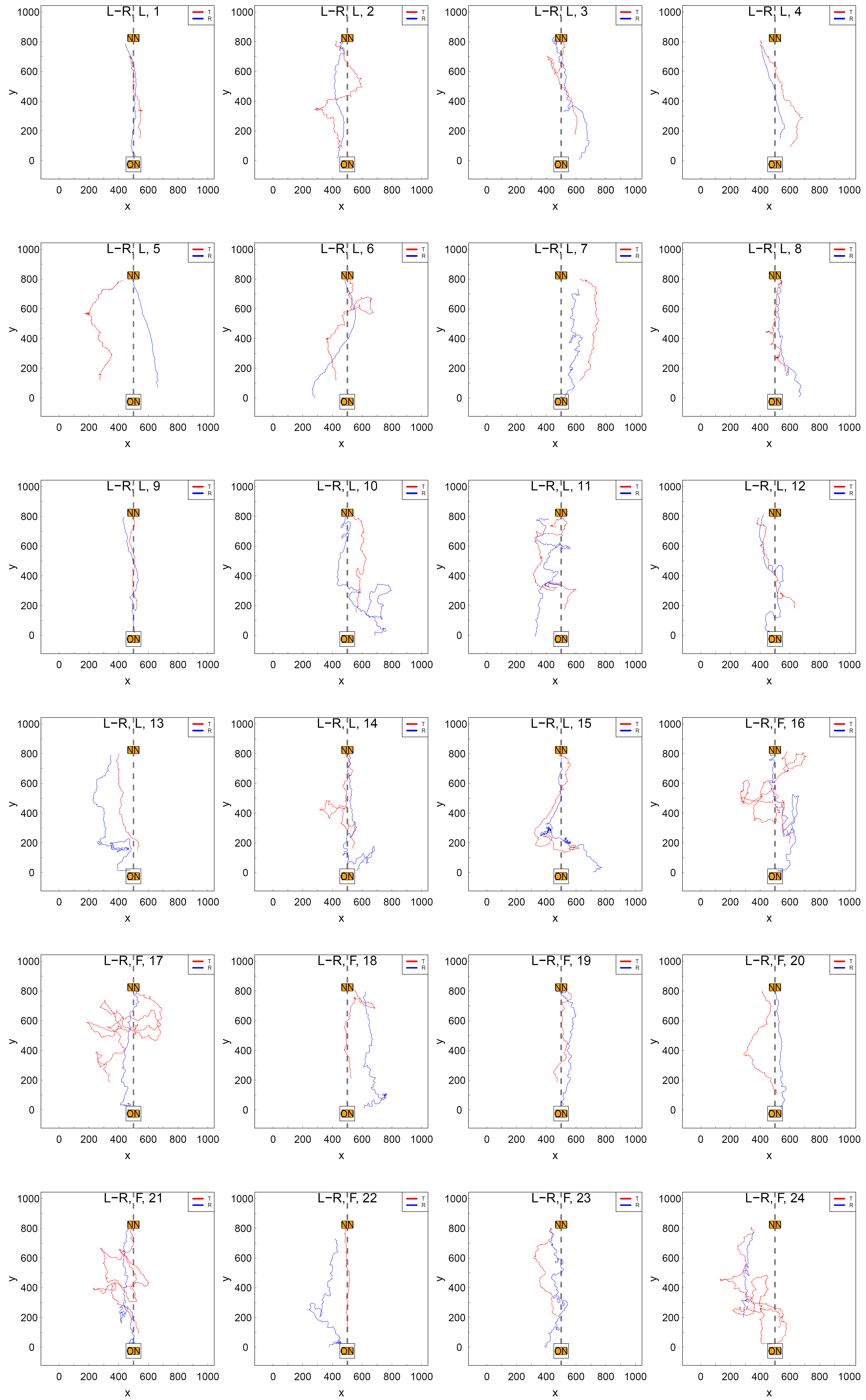


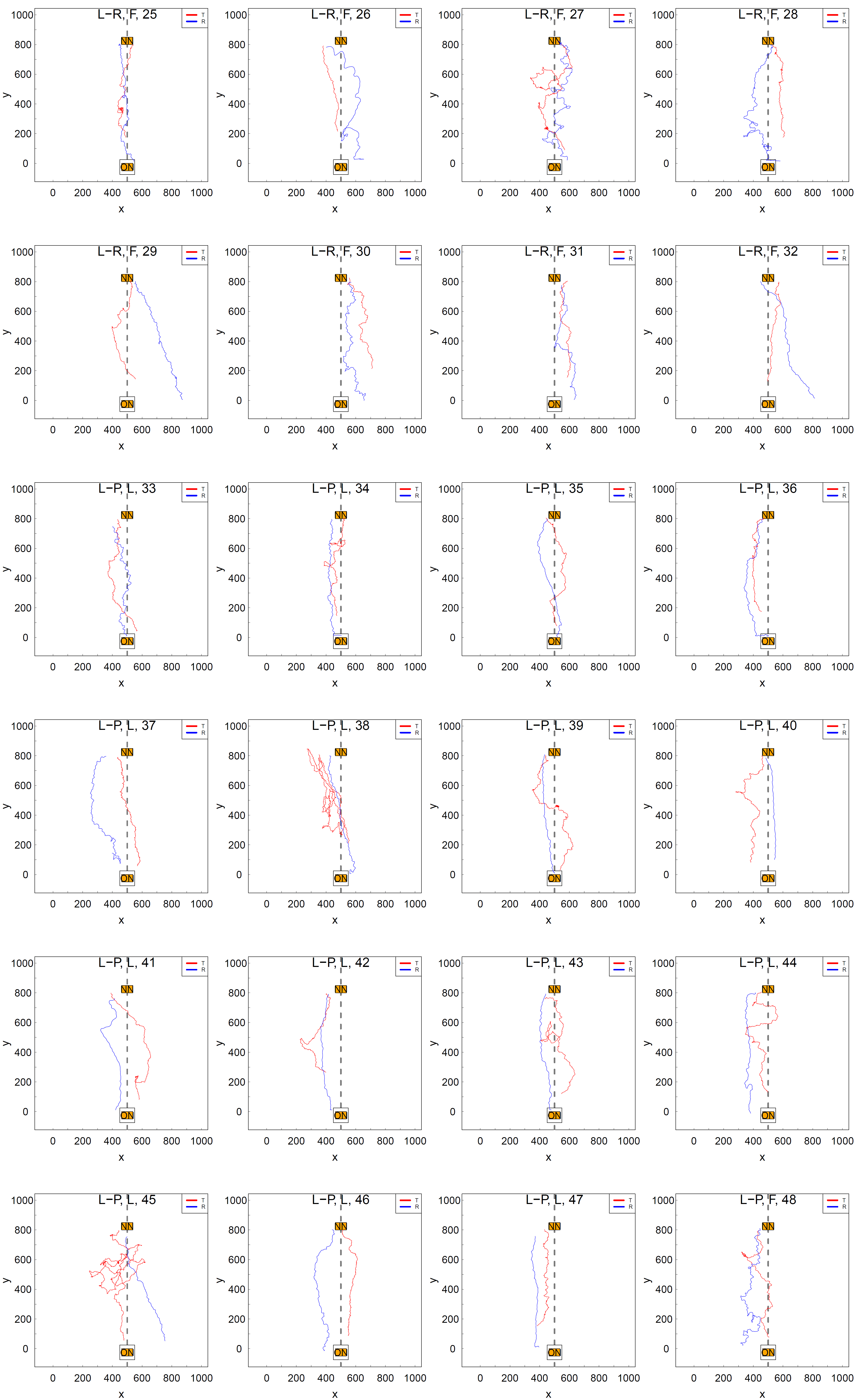


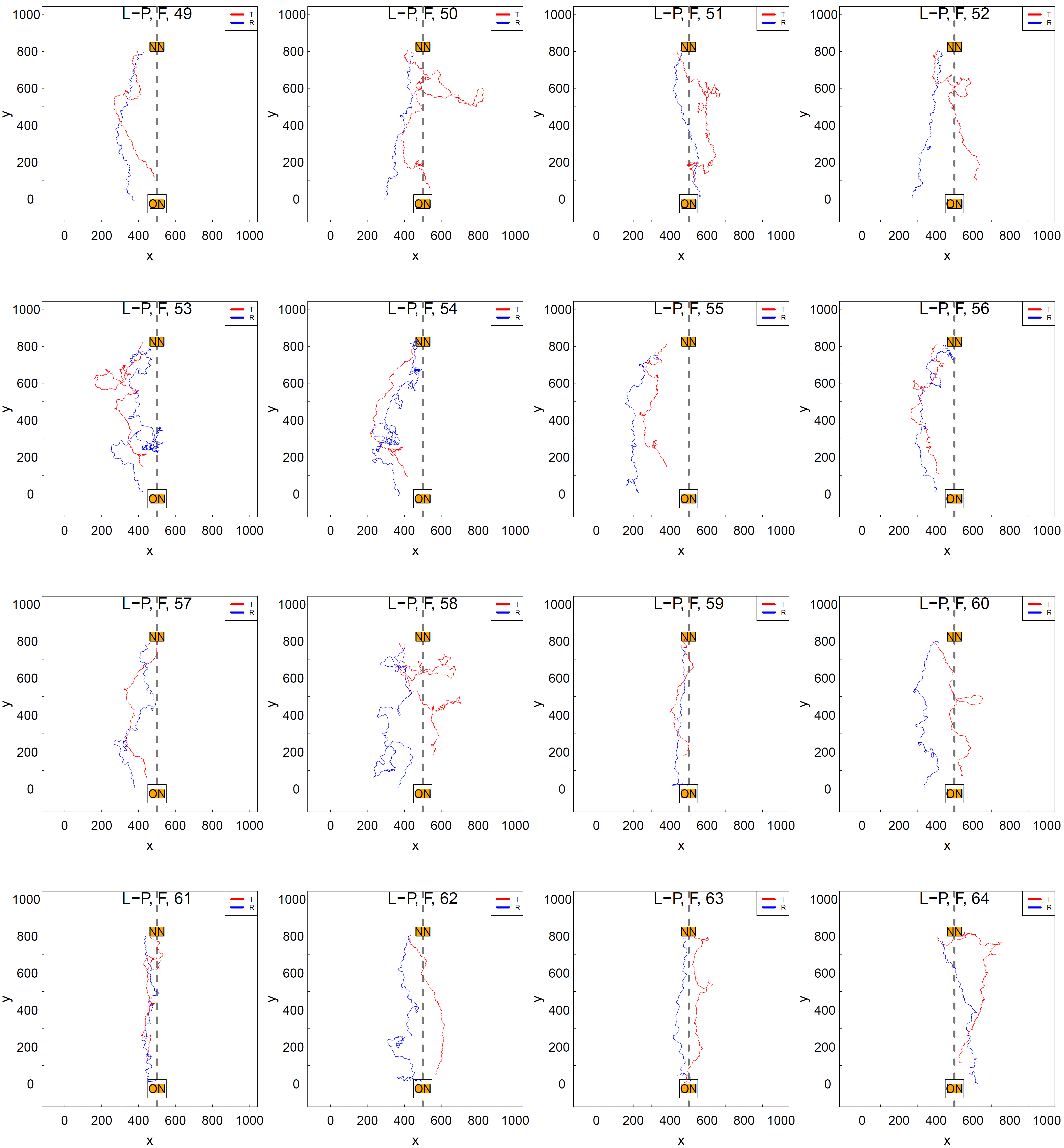


**Fig. S5** The paired Tandem path (red) and Return path (blue) for each Leader (L) and Follower (F) in the Landmark-reach (L-R) treatment and the Landmark-poor (L-P) treatment ON indicates the old nest with exit at x=500 mm, y=0 mm. NN indicates the new nest with exit at x=500 mm, y = 800 mm. The black rectangle around the ON indicates the Petri dish covering the ON during the tracking of a tandem or return journey by the gantry. The interrupted grey line indicates the midpoint along the x-axis, x = 500 mm. When identifying paths with loops, we ignored any loops touching the ON or the Petri dish covering the NN.

**Fig. S6** Distance (mm) between the paired Tandem and Return paths for Leaders (L) and Followers (F) for each of the two treatments: Landmark-reach (L-R) and Landmark-poor (L-P). The distance was measured as the mean of the nearest neighbour distances between the points on the paired Tandem and Return paths (for more details see Methods). The data points are superimposed over the distributions represented by the boxplots. Note the log scale for Distance (mm). For more details on symbols, please see the caption for Fig. 4 in the main text.
